# Optimal Experimental Design to Estimate Statistically Significant Periods of Oscillations in Time Course Data

**DOI:** 10.1371/journal.pone.0093826

**Published:** 2014-04-03

**Authors:** Márcio Mourão, Leslie Satin, Santiago Schnell

**Affiliations:** 1 Department of Molecular and Integrative Physiology, University of Michigan Medical School, Ann Arbor, Michigan, United States of America; 2 Department of Pharmacology, University of Michigan Medical School, Ann Arbor, Michigan, United States of America; 3 Department for Computational Medicine & Bioinformatics, University of Michigan Medical School, Ann Arbor, Michigan, United States of America; 4 Brehm Center for Diabetes Research, University of Michigan Medical School, Ann Arbor, Michigan, United States of America; Universitat Pompeu Fabra, Spain

## Abstract

We investigated commonly used methods (Autocorrelation, Enright, and Discrete Fourier Transform) to estimate the periodicity of oscillatory data and determine which method most accurately estimated periods while being least vulnerable to the presence of noise. Both simulated and experimental data were used in the analysis performed. We determined the significance of calculated periods by applying these methods to several random permutations of the data and then calculating the probability of obtaining the period's peak in the corresponding periodograms. Our analysis suggests that the Enright method is the most accurate for estimating the period of oscillatory data. We further show that to accurately estimate the period of oscillatory data, it is necessary that at least five cycles of data are sampled, using at least four data points per cycle. These results suggest that the Enright method should be more widely applied in order to improve the analysis of oscillatory data.

## Introduction

Physiological rhythms are essential to life. However, they can be difficult to observe experimentally due to the natural stochastic fluctuations exhibited by most physiological systems, and the random or irregular noise present in the experimental measurements themselves. Methods for detecting pulsatility and estimating the period of oscillations are very important in modern biology and require the integration of statistical, mathematical and experimental approaches.

The analysis of pulsatility in the biomedical sciences is generally done using widely accepted methodologies such as ULTRA, Cluster and PulseFit [1]–[3]. However, the estimation of oscillatory periods requires a different set of methods than those used to detect pulsatility. Methods to estimate the period of oscillations have been extensively discussed over the past two decades; they include the Whittaker-Robinson periodogram [4]–[7] that was popularized to study biological time course data by Enright [8]–[10], Fourier spectral analysis [4], [6], [7], [9], Lomb-Scargle periodogram [10], MESA [4], [5], [7], Autocorrelation [11] and cosinor [9], [10], [12]. All these methods are valid under different assumptions and may provide different results when applied to the same time course.

In a seminal paper, Refinetti [13] investigated the accuracy and noise tolerance of six different methods for estimating circadian periods (24 hours): Enright's periodogram, Fourier spectral analysis, Autocorrelation, acrophase counting, inter-onset averaging, and linear regression of onsets. Using *in silico* generated circadian rhythm datasets consisting of cosine and square waveforms, he found that Enright's periodogram and Fourier analysis outperformed the other methods in estimating circadian rhythms. Refinetti also found that Enright's periodogram and periodic Autocorrelation exhibited a higher noise tolerance.

Period estimation is insufficient without determination of its statistical significance. This is especially true if the data contains high levels of noise as there can be high probability of error. The estimation of statistically significant oscillatory periods is the subject of some controversy, in part due to the association of these methods with theoretical false alarm functions [14], [15]. False alarm functions state the probability of obtaining a power in the periodogram that is greater than some power of reference and are used to evaluate the significance of periodogram peaks. However, as these functions are only applicable under limited conditions, they often fail to provide an adequate measure of the probability of obtaining a particular period [16]. For example, the χ^2^ theoretical cumulative distribution is commonly used to attach significance to a period in the Enright periodogram. Nevertheless, it is shown to be only applicable with a minimum number of 10 blocks of data collected with 2400 data points (a sample frequency equal to one point every 6 minutes) in determining the period of circadian rhythms [13], [17]. In spite of these limitations, a sampling frequency of 1 hour [18] or 24 hours [19] has been presented as sufficient to correctly estimate the rhythms using the Enright periodogram.

The design of experiments to estimate oscillation periods has been discussed previously [6], [7], [13]. The effects of sampling frequency, the number of cycles and noise in the time course data have been considered. For example, Refinetti [13] found inaccurate estimates are obtained from a time series having a low density sample of points. The general recommendation is that the time course data should be collected with high sampling frequency and number of cycles in order to obtain good estimates of oscillation periods. However, to date, the minimum number of data points and cycles in the time course needed to obtain statistically significant oscillation periods remains to be determined. This requires a systematic investigation of the ideal characteristics of the experimental time course data, and it would be of great importance for experimentalists for this to be established.

The aim of the present paper is to compare how well the Autocorrelation [20]–[23], the Enright periodogram [8], and the Discrete Fourier Transform (DFT) method [20], [24], [25] estimate the significance of periods in oscillatory time course data. The Autocorrelation method, also called the periodic Autocorrelation, is used to quantify randomness by computing autocorrelations for data values at varying lags in a course data. Autocorrelations are near zero for randomness [26]. The Enright periodogram method compares the variance of data within blocks of different sizes with the total variance of the data [8]. The DFT analyzes how well sinusoidal waves of different frequencies fit a particular course data.

**Figure 1:**
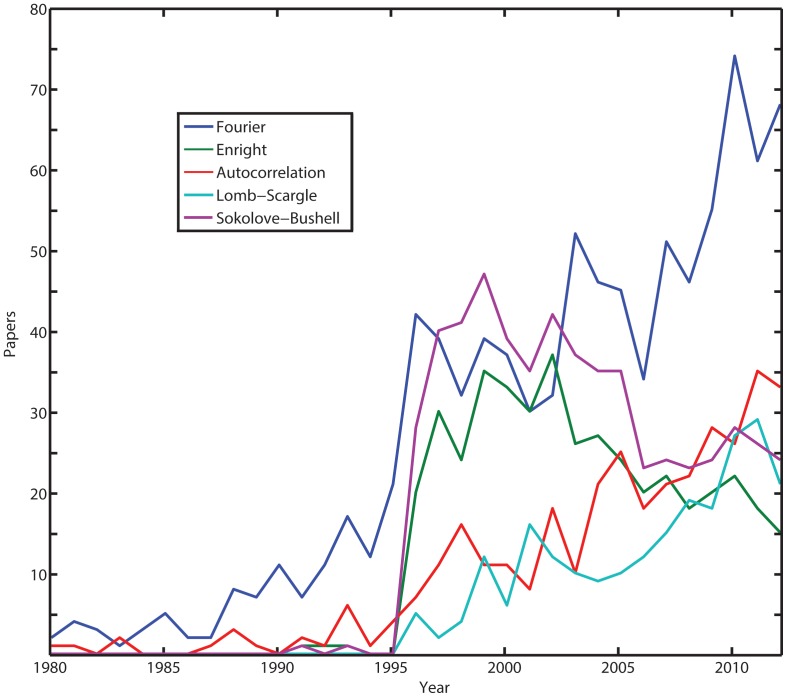
Citations to Discrete Fourier Transform, Autocorrelation method, Enright periodogram, Lomb-Scargle periodogram and Sokolove-Bushell periodogram for the last 30 years. The figure was drawn from data in Scopus, checked in October 1st 2013. The search was limited to the subject areas of “Life Sciences” and “Health Sciences”. The search shows that the DFT and Autocorrelation methods are the most widely cited approaches and are on the rise. The Enright periodogram usage seemed to start declining once papers using the Lomb-Scargle periodogram started to increase. The search was performed using the keywords: “Oscillations”, “Rhythm” OR “Rhythms” in the article title, abstract or keywords; “method” in “All fields”; “Discrete Fourier Transform” OR “Fast Fourier Transform”, for the FFT or DFT methods; “Autocorrelation method”, for the Autocorrelation method; “Enright periodogram” OR “chi-square periodogram”, for the Enright method; “Lomb periodogram” OR “Lomb-Scargle periodogram” OR “Lomb” AND “Scargle”, for the Lomb periodogram; “Sokolove-Bushell periodogram” OR “Sokolove-Bushell” OR “Sokolove” AND “Bushell”, for the Sokolove-Bushell method.

We focus our analysis on these particular methods because they are the most commonly used approaches in the life science literature over the last 30 years. A Scopus search shows that citations of the DFT and Autocorrelation method are on the rise, while the Enright periodogram has only recently started to decline after the Lomb-Scargle periodogram appeared in the literature (see, Fig. 1). In our analysis, we use simulated as well as real data to help design experiments that can yield useful estimates of significant periods. We evaluate the accuracy of the oscillatory periods we obtained by simulating data having different numbers of cycles, sampling frequencies and noise. In addition, instead of relying on theoretical false alarm probability functions, we compute the significance of oscillatory periods by computing random permutations of the time course data. By combining our analysis of experimental design and analytical methods to estimate the significant periods of rhythms, we determined both the ideal method to investigate rhythms and the minimal number of cycles and data points required to properly estimate oscillation periods that have adequate statistical significance.

## Methods

One of the first steps in designing an experiment to estimate the period of an oscillation is to choose an appropriate number of cycles and points per cycle to be measured. Simulation of time course data can help the experimental biological rhythm scientist to design experiments.

### Simulations of time course data

For rhythms with one period, we generated oscillatory data using the sine expression:
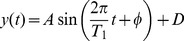
(1)


For a single simulated experiment, *y(1), y(2), …, y(n)* are the *N* data points at times *_t1, t2, …, tn_*. All simulations have a nominal amplitude *A = 1*, phase *φ = 0* and center amplitude *D = 0*. The period *T* of oscillations is equally spaced between 1 and 20 *(1, 2, 3, …, 20)*. To investigate how the experimental design affects the estimation of the period *T*, we simulated a set of time courses by varying the number of cycles (*NC*) or number of times a period *T* is repeated, and the number of points per cycle (*NPC*) in the time course data. Note that the total number of points *N* in a time course data is *N = NC·NPC*. A typical mono-oscillatory time course is shown in Supplemental Fig. 1A. We generate a set of simulated mono-oscillatory data using *2≤NC≤20* and *3≤NPC≤20*. This produces a total number of 6840 time courses for evaluation.

For oscillations containing two periods (a fast and a slow period), we generated oscillatory data using the following expression:
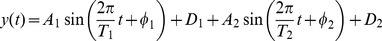
(2)


For a simulated experiment, *y(1), y(2), …, y(n)* are the *N* data points at times *t1, t2, …, tn*. In the above equation, the period of the oscillations *T_1_* and *T_2_* is spaced between 1 and 20 *(1, 2, 3,…, 20)*. As with the mono-oscillatory data, all simulations have nominal amplitude *A = A_1_ = A_2_ = 1*, phase *φ = φ_1_ = φ_2_ = *0 and center amplitude *D = D_1_ = D_2_ = 0*. Also, the time course data is bound between *2≤NC≤20*. However, for the bi-oscillatory data, *NC* corresponds to the number of repetitions of the slowest period in the time course, while *NPC* is the number of points of the fast period in the time course. We simulated a total of 64,980 time courses for evaluation. A typical bi-oscillatory time course is shown in Supplemental Fig. 1B.

For each time course, we introduced noise to account for the experimentally observed error in the time course measurements. For example, for the mono-oscillatory time course, data with Gaussian distributed random error can be modeled by 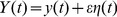
(3)


In this expression, *Y(t)* is the course data with random error, *ε* represents the strength of the noise, and *η(t)* is the Gaussian distributed error with mean zero and standard deviation one.

### Autocorrelation method

The Autocorrelation method is used to quantify randomness of a time course by computing autocorrelations for data values at varying lags in the data. It has been widely used to recover biological oscillatory periods [20]–[23]. The lag *k* autocorrelation function is defined as:
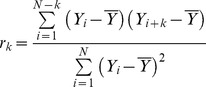
(4)


If there is an oscillatory period with lag *k* in the time course data, *r_k_* is approximately equal 1. Autocorrelations will be around zero for a random time course [26]. To analyze a time course data, we construct the Autocorrelation plot by calculating *r_k_* values for all possible *k* lags, where 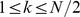. The minimum period corresponds to the distance between two data points. We calculate the local maxima of the Autocorrelation plot and sort by ascending all periods with *r_k_* values higher than 10% the maximum *r_k_* value estimated from the Autocorrelation plot. Because we can only assess the significance of a limited number of peaks, we use 10% as an arbitrary threshold to prevent small peaks in the periodogram from being included in the period estimation analysis. Since the Autocorrelation formula does not identify harmonics in the time course data, we remove every multiple of the periods found in the previous step.

### Enright Periodogram

We implement the method first described by Enright [8] using the statistic 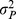 formula:
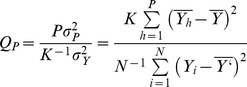
(5)


In the above expression *P* is the number of points in one block of data, 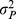 is the variance of the data comprised of *P* points, *K* is the number of blocks of size *P*, 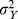 is the variance of the full course data, and *N* is the total number of points in the data. If the time course data of *N* number of points exhibits oscillations with period *P*, the ratio 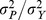 is approximately equal to 1.

For a particular time course data, we construct the Enright periodogram by calculating *Q_p_* values for all possible blocks of *P* points, where 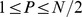. The minimum period corresponds to the distance between two data points. We calculate the local maxima of the periodogram and sort by ascending period all periods with *Q_p_* values higher than 10% the maximum *Q_p_* value found on the periodogram. Note that we can only assess the significance of a limited number of peaks. We use 10% as an arbitrary threshold to prevent small peaks in the periodogram from being considered in the period estimation analysis. As the Autocorrelation method, the Enright method does not identify harmonics in the course data. Hence, we remove every multiple of the periods found in the previous step.

### Discrete Fourier Transform

The Fourier method analyzes how well sinusoidal waves of different frequencies fit a particular course data [27]. The Discrete Fourier Transform at a frequency *ω* can be obtained using the following equation:
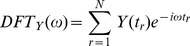
(6)


We estimate the power spectral density of the signal by the function 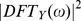. This function is the classical periodogram [14] and follows the definition originally given by Shuster [28].

Let us consider the above expression. We assume that the time course data oscillates with a frequency equal to *ϖ*. If the estimate frequency *ω* is significantly different from *ϖ*, the components *Y(t_r_)* and 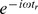
are out of phase and the product oscillates rapidly. In this situation, the sum will have a value close to zero. On the other hand, if the frequency *ω* is very close to *ϖ*, the components *Y(t_r_)* and 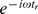
are in phase and their product oscillates rapidly. The sum of the products will produce a maximum peak or power when *ω* is equal to *ϖ*.

For a particular course data, we calculate the frequency spectra by evaluating Eq. (6) on a range of sampled frequencies. Here, the minimum frequency is given by 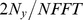, where *N_y_* is the Nyquist frequency (half of the sampling frequency) and *NFFT* is the next highest power of 2 greater than the length of the course data. The maximum frequency is half the *NFFT* multiplied by *y*. We calculate the local maxima of the frequency spectra and sort first by the highest power all frequencies with power values higher than 10% the maximum power value found. As we mentioned before, we can only assess the significance of a limited number of peaks. We decided to use 10% as an arbitrary threshold to prevent small peaks in the frequency spectra from being considered in the period estimation analysis. The DFT method identifies harmonics in the time course data, so we did not need to remove multiples of the periods found as we did for the Autocorrelation and Enright methods.

### Calculation of the relative difference

The relative difference (*RD*) is used to calculate the accuracy of a particular method in the estimation of a simulated period in the oscillatory data. It is obtained using the following mathematical expression:
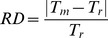
(7)where *RD* is the relative difference, *T_m_* is the period returned by the method and *T_r_* is the period used to generate the data.

### Computer implementation of algorithms and data analysis

In this work, the algorithms were developed using MATrix LABoratory (MATLAB) (Ver. R2012b). Simulations were primarily conducted on a high performance computing Flux (AMD Opteron/Intel Nehalem 64-bit) cluster at the University of Michigan Center for Advanced Computing, which has over 5,000 cores with an average of 2GB RAM/core to analyze the performance of three main methods in the estimation of oscillatory periods.

In all methods, the maximum period to analyze is set to half the maximum range. As a consequence, we only evaluate periods that repeat at least twice in the data. The minimum period and the distance between evaluated periods depend on the method applied. For each time course evaluated, we restrict the evaluation of significance to five candidate periods. The inclusion of more candidates does not lead to results with higher accuracy. These are the first five periods with a maximum in the Enright and Autocorrelation periodogram and the five periods with the highest maximum in the DFT frequency spectrum. The significance of a candidate period is determined by calculating the probability of obtaining the power values exhibited in the periodogram and frequency spectrum. For this purpose, we analyze 10,000 random permutations of the course data. A period is considered significant if the number of power values (*r_k_*, *Q_P_* or *DTF_y_(ω)* obtained by the random permutations greater than the power value associated with the period (p-value) occurs less than 1%(level of significance) of the times.

Matlab codes and sample data is available in the **Supplementary Information**.

## Results

We used oscillatory data collected in experiments for which periods have been established (and published) to check if our algorithm performs as expected and recovers with significance the correct period. For this purpose, we applied the Enright periodogram, DFT and Autocorrelation methods to recordings of pancreatic islet free calcium oscillations, a physiologically important variable that helps regulate the secretion of insulin [29]. The candidates used in the Autocorrelation and Enright methods were selected by obtaining the local extrema of resulting periodograms and then ordering them by period. The analysis of significance was restricted to the first five non-multiple periods, where the values of the function corresponded to local extrema in the periodogram. In the DFT method, after obtaining the extremum, we searched for the first five highest peak candidates (see **Methods** for more details).

**Figure 2:**
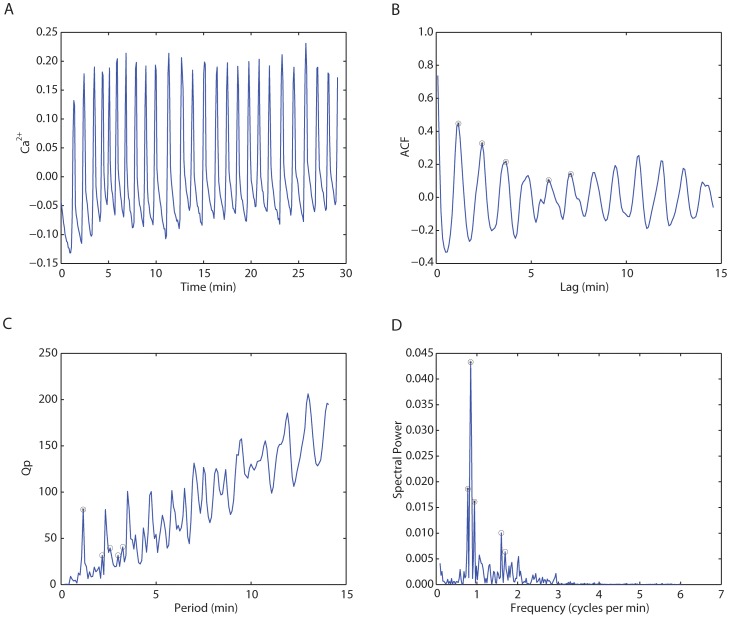
All three methods, Autocorrelation, Enright and DFT predict approximately 1.17 minute periods in islet Ca^2+^ oscillations. (A) 30 minutes of oscillatory data representing Ca^2+^ oscillations; (B) Autocorrelation periodogram generated with the Autocorrelation method. The plot shows 5 significant local maxima obtained at the significance level of 0.01; (C) Enright periodogram generated by the Enright method. The plot shows 1 significant and 4 non significant local maxima obtained at the significance level of 0.01; (D) Frequency spectrum generated with the DFT method. The plot shows 3 significant and 2 non significant oscillatory periods obtained at the significance level of 0.01. Closed circles represent significant periods. Open circles represent non significant periods. We selected a maximum of five oscillatory candidate periods in each method. The candidates were ordered by the value of its period in the Autocorrelation and Enright methods, and by power in the DFT method.

Fig. 2A shows the Ca^2+^ oscillations of a pancreatic islet monitored for 30 minutes. Both the Autocorrelation (Fig. 2B) and Enright (Fig. 2C) methods identified a significant period in the data of 1.17 minutes, while the DFT method identified a period of 1.18 minutes (see Fig. 2D). This value is in agreement with the reported periods of fast pancreatic islet calcium oscillations, and from simple visual inspection of the data shown [29]. While both the Autocorrelation and Enright methods predicted the period correctly as the lowest significant period found in the data, DTF (Fig. 2D) identified the correct period as the highest significant peaks in the frequency spectrum (see Fig. 2B,C,D). The methods also identify the periods of the remaining islets with high accuracy (results not shown) [29].

### The optimal candidate period is the period whose peak is associated with the minimum p-value

There are two different criteria that can be used to choose the optimal candidate period. On one hand, one can simply choose the highest maximum of the periodogram or frequency spectrum. On the other hand, one can choose the local maximum which occurs least often in randomizations of the data. These two criteria are not necessarily the same, especially when significant levels of noise are present. To identify which criterion (maximum peak or minimum p-value) is most likely to yield the correct period, we compared the simulated period used to generate each set of time course data with five candidate periods obtained using all three methods (see **Methods**). We chose the best candidate period as that having the maximum peak or the minimum p-value. We then calculated the *RD* of the best candidate period to the simulated period (see **Methods**).

**Figure 3:**
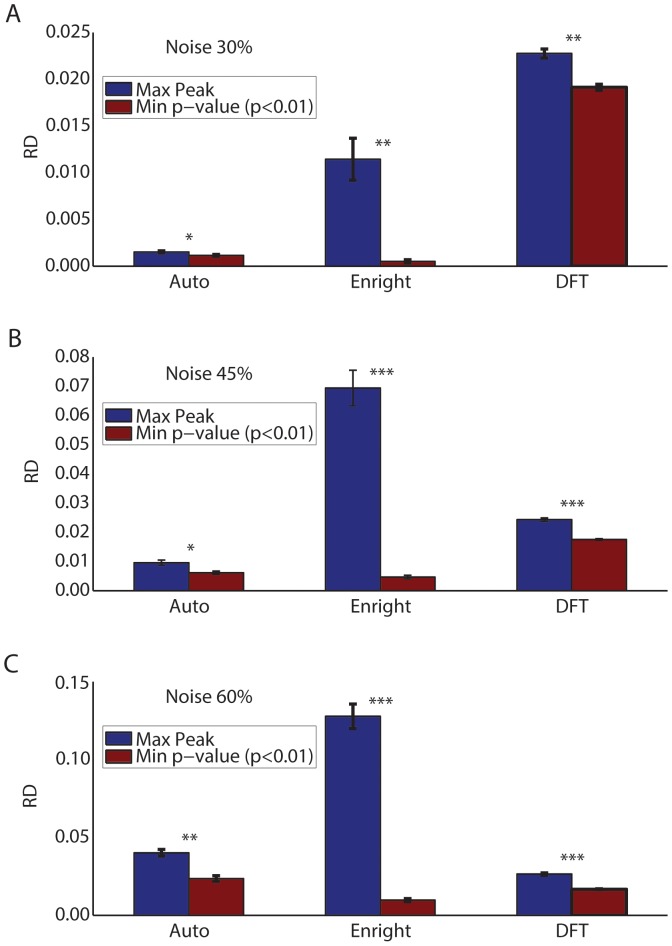
Average relative difference (*RD*) obtained choosing the candidate period as either the maximum peak (blue bars) or the minimum significant p-value (red bars) in *in silico* mono-oscillatory data for all three methods. (A) Results obtained with 30% noise; (B) Results obtained with 45% noise; (C) Results obtained with 60% noise. In all plots, candidate periods with the minimum significant p-value (level of significance is set to 0.01) provide higher accuracy results than candidates with the maximum peak. We performed t-tests to check whether the maximum peak and the minimum significant p-value approach provide significantly different outcomes: (A) From left to right, p-values are 0.0245, 1.3272×10^−6^ and 2.3793×10^−10^, (B) From left to right, p-values are 0.0013, 5.1381×10^−26^ and 3.6033×10^−25^, (C) From left to right, p-values are 6.3398×10^−09^, 1.0977×10^−49^ and 8.93×10^−35^. We denote *, ** and *** as p-values lower than 0.05, 1×10^−5^ and 1×10^−10^, respectively. In each plot, from left to right, pairs of bars represent the Autocorrelation, Enright and DFT method.

As shown in Fig. 3, on average, the candidate period having the minimal p-value (red bars) provided higher accuracy (lower *RD* values) than the candidate period having the maximum peak (blue bars). This was true for all methods applied to mono-oscillatory data where different levels of noise were present, but was especially noticeable when we used the Enright method (middle bars). With 30% noise, all of the methods tested displayed high accuracy (i.e. corresponding to relative differences lower than 5%) (see Fig. 3A). However, as expected, accuracy decreased with higher noise values. Using the minimum p-value (red bars), values close to 0.25% in data sets with 30% noise increased to values near 1% with 45% noise and near 2% with 60% noise for both the Autocorrelation and the Enright methods. The DFT was largely insensitive to noise and displayed values close to 3% for data that had different noise levels. Overall, all of the methods were found to be highly accurate, but the minimum significant p-value approach provided more accurate and significantly different estimates of the true periodicity of the data.

**Figure 4:**
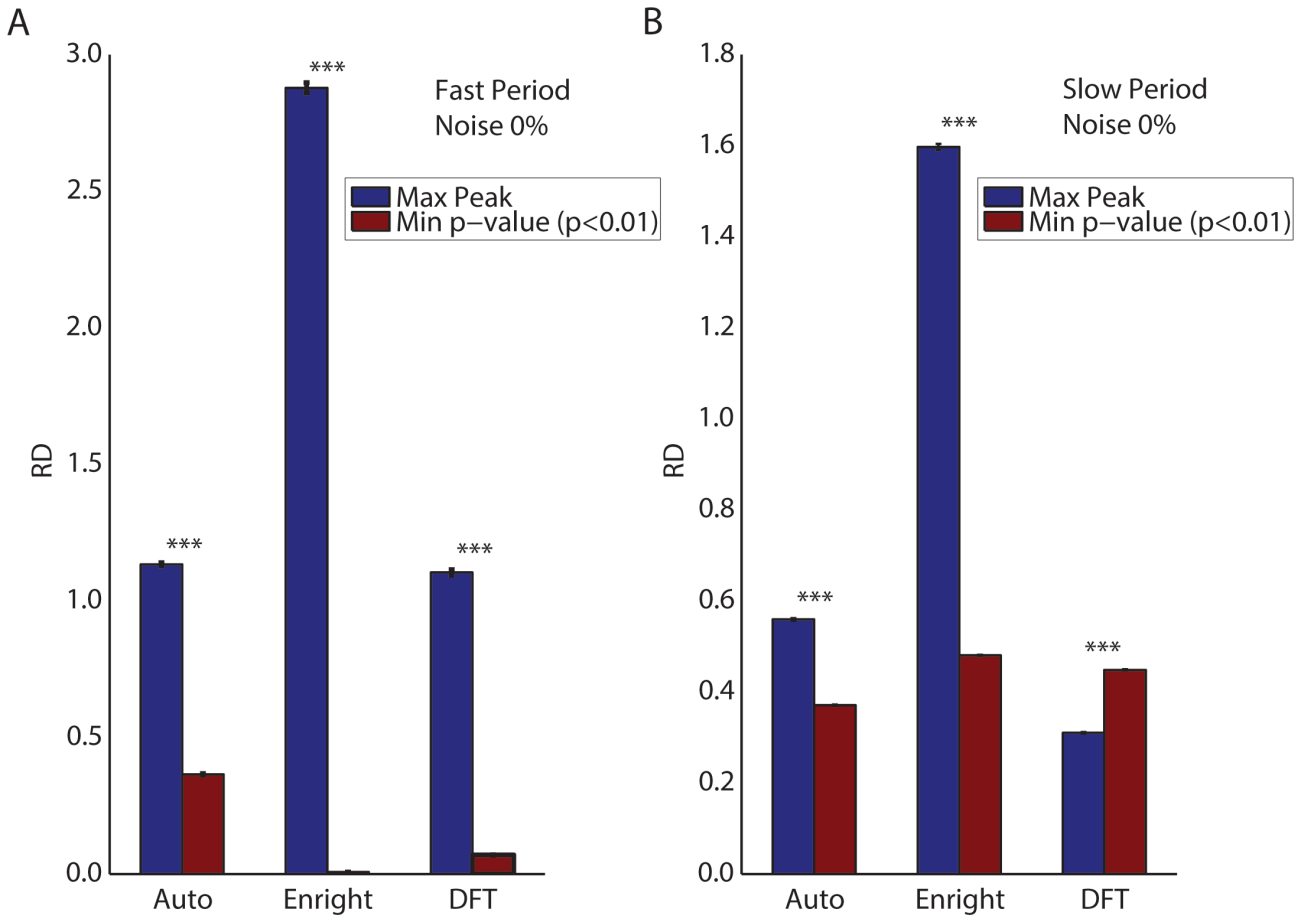
Average relative difference (*RD*) obtained choosing the candidate period as either the maximum peak (blue bars) or the minimum significant p-value (red bars) in *in silico* bi-oscillatory data for all three methods. (A) Results obtained with 0% noise for the fast period. Candidate periods with the minimum significant p-value (level of significance is set to 0.01) provide higher accuracy results than candidates with the maximum peak. (B) Results obtained with 0% noise for the slow period. Similarly to the fast period, candidate periods with the minimum significant p-value (level of significance is set to 0.01) provide higher accuracy results than candidates with the maximum peak, with exception of the DFT method. We performed t-tests to check whether the maximum peak and the minimum significant p-value approach provide significantly different outcomes. p-values are all equal to 0. We denote *** p-values lower than 1×10^−10^. For each plot, from left to right, pairs of bars represent the Autocorrelation, Enright and DFT method.

We performed a similar analysis using bi-oscillatory data and then compared the results obtained using either the maximum peak or the minimum significant p-value to select the optimal candidate period (Fig. 4). The minimum p-value (red bars) was clearly more accurate (lower RD values) in estimating the fast oscillatory period (Fig. 4A). In fact, all of the methods were poor in predicting the fast simulated period using candidate periods with the maximum peak (Fig. 4A, blue bars). This suggests that the candidate periods identified using the maximum peak are not a good choice for predicting the periods of fast oscillatory data (see **Discussion**). The accuracy using the minimum p-value decreases for the slow simulated period of the bi-oscillatory data (see Fig. 4B). This approach provides higher accuracy using the Autocorrelation and Enright methods, but not the DFT method. Overall, while predicting the slow simulated period, all of the methods we tested produced inaccurate results. In the best case, selecting the period using the maximum peak produced relative differences close to 30% (Fig. 4B).

### Increasing the number of cycles collected improved the accuracy of the analysis of mono-oscillatory data

**Figure 5:**
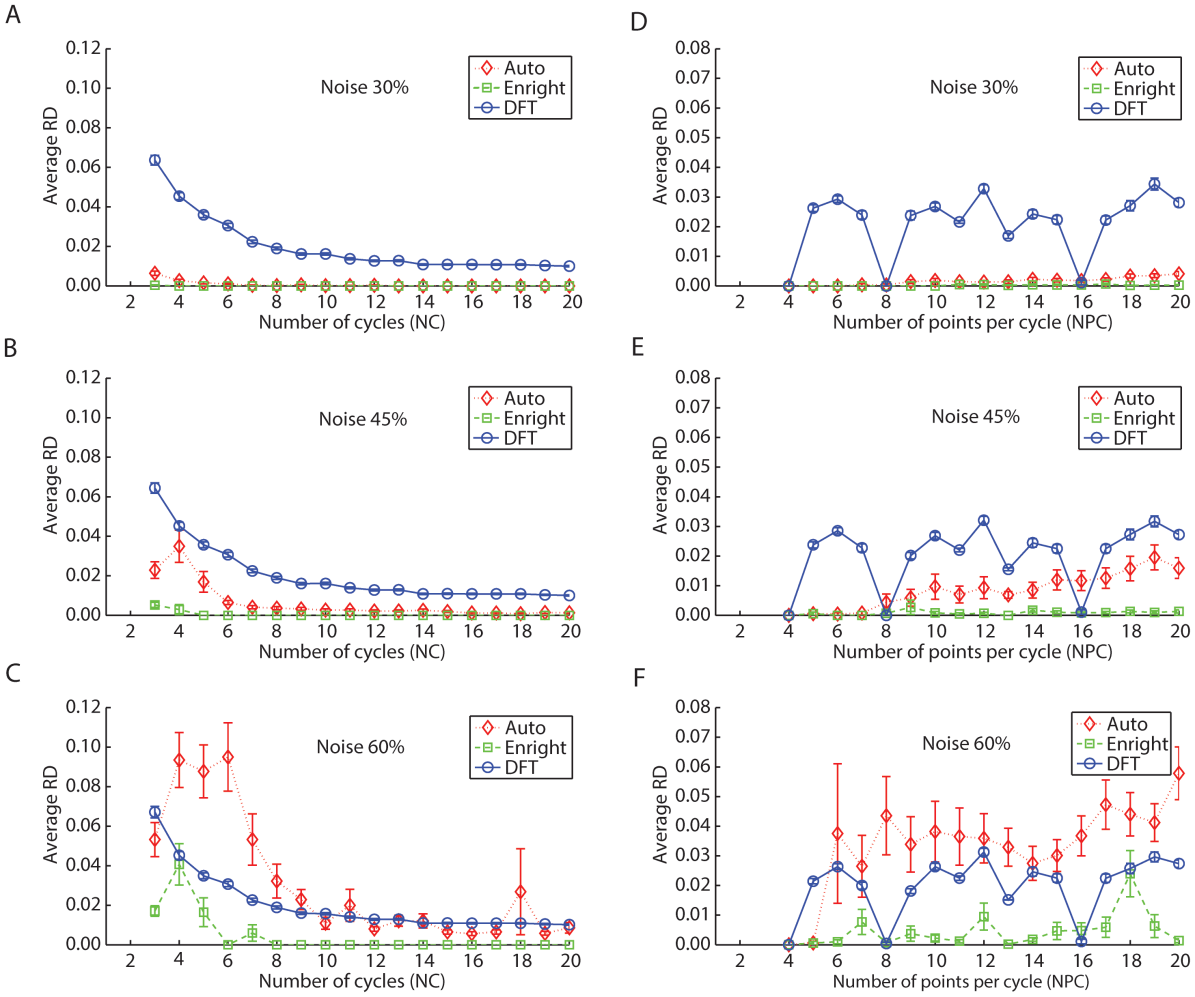
Autocorrelation, Enright and DFT method's average and standard error relative difference as a function of the number of cycles (*NC*) (left) and the number of points per cycle (*NPC*) (right) in *in silico* mono-oscillatory data. (A, D) Results obtained with 30% noise. (B, E) Results obtained with 45% noise. (C, F) Results obtained with 60% noise. The Enright method outperforms both Autocorrelation and DFT methods, where at least 5 repetitions of an expected period are necessary in order to reduce the relative difference to values less than 5%; The Enright and the DFT method's performance remain invariant with *NPC*. The Autocorrelation method's performance worsens with increasing *NPC*. The red diamond, the green square and the blue circle represent the Autocorrelation, Enright and DFT methods, respectively.

To evaluate the effects of changes in the number of cycles (*NC*) and the number of data points sampled per cycle (*NPC*) in the estimation of periods, we compared the period used to generate the data (simulated period) with the optimal candidate period. Here, we used the minimum significant p-value as the optimal candidate period since this criterion guaranteed higher accuracy (see previous section). As previously, we calculated the difference between the simulated and estimated period to obtain an average *RD* (see **Methods**) as a function of *NC* and *NPC* (Fig. 5).

With 30% noise present in the data, both the Autocorrelation and Enright methods accurately predicted the simulated period; note the small error obtained in Fig. 5A. However, as the levels of noise increased (60%) the accuracy of these two methods decreased. This contrasts with DFT performance which remained relatively unchanged despite increasing levels of noise (Fig. 5). The average *RD* of the simulated period of all three methods thus diminished as *NC* increased, with at least 5 cycles being necessary to reduce *RD* to less than 5%. Overall, the Enright method performed the best, exhibiting differences in the simulated period of less than 1%, with at least 7 *NC* were analyzed in data sets having 60% noise (Fig. 5C).

While the *RD* decreased with increasing *NC*, it was relatively unchanged with *NPC*, suggesting that simply increasing the number of data points does not help reduce the error. The only exception occurred using the Autocorrelation method, where the *RD* increased with increasing *NPC*, while overall the average difference was less than 5% for all of the methods (see Fig. 5E, F and **Discussion**). Among the five candidate periods analyzed in every set of time course data, at least 4 *NPC* were necessary to obtain significant results. To make sure these results were independent of the simulated period, we calculated the variation across periods of the relative differences for all possible combinations of *NC* and *NPC*. The mean value for all combinations with 60% noise was 0.0047, 0.0065 and approximately 2.41×10^−4^ for the Autocorrelation, Enright and DFT methods, respectively.

### The Enright method produced the most accurate results in the estimation of fast periods of bi-oscillatory data

**Figure 6:**
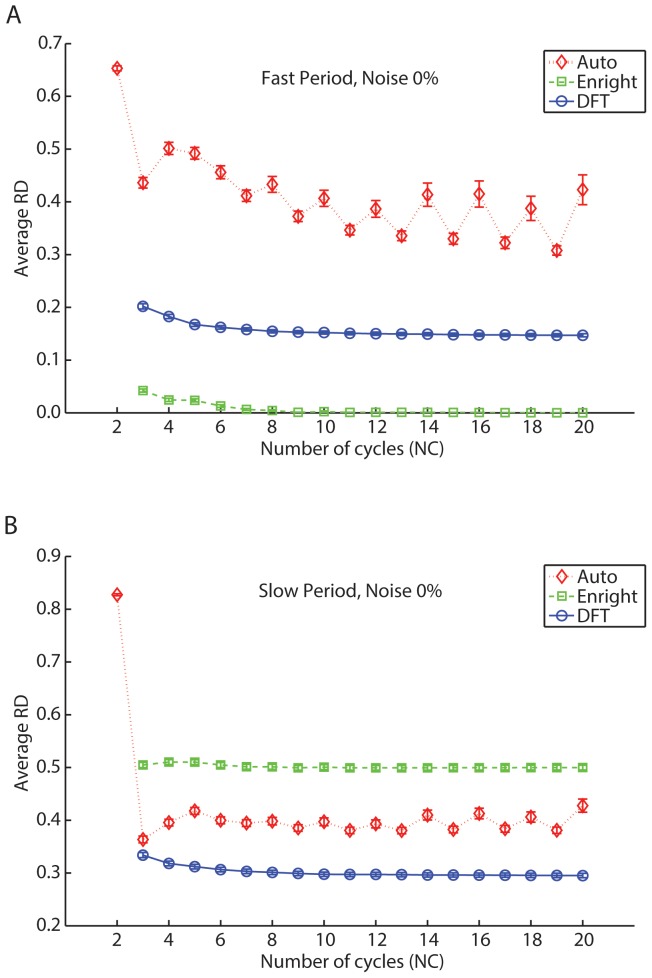
Autocorrelation, Enright and DFT method's average difference (*RD*) and standard error in recovering the fast and the slow period of *in silico* bi-oscillatory data as a function of the number of cycles (*NC*), with 0% noise. (A) Both Enright and DFT method's relative differences (*RD*) slightly decrease with increasing *NC* while recovering the fast simulated period. The Enright method returns on average, better results than the DFT method, where at least 3 *NC* produce average differences less than 5%. The Autocorrelation method is the least accurate, producing average differences of around 40%. It also remains fairly constant with increasing *NC*. (B) The Autocorrelation method produces the most accurate results, but overall, average differences surpass the 35%. The red diamond, the green square and the blue circles represent the Autocorrelation, Enright and DFT methods, respectively.

**Figure 7:**
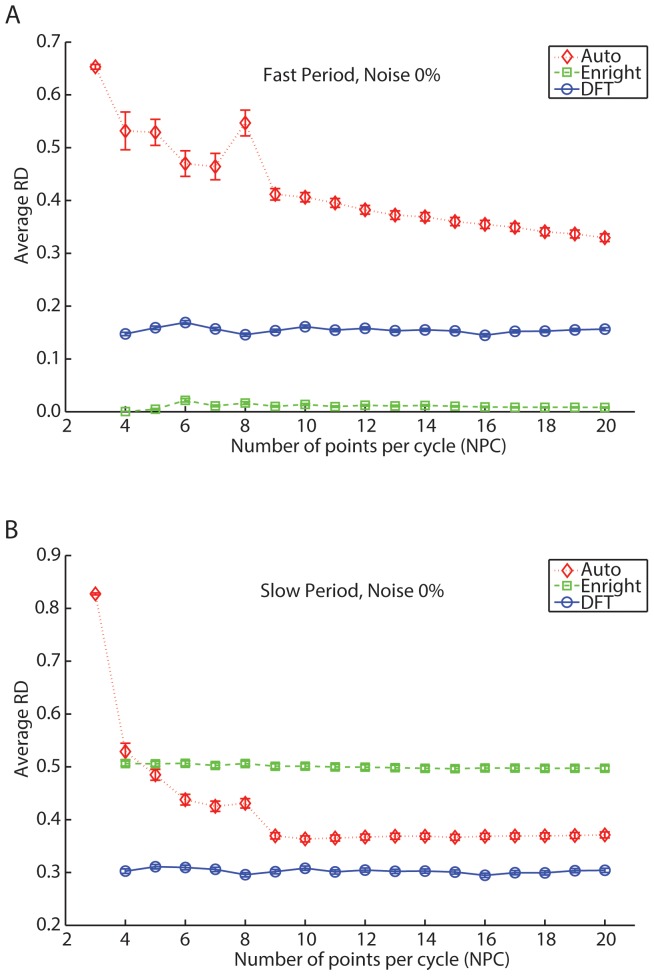
Autocorrelation, Enright and DFT method's performance in recovering the fast and the slow period of *in silico* bi-oscillatory data as a function of the number of points per cycle (*NPC*), with 0% noise. (A) Both Enright and DFT method's relative differences (*RD*) remain fairly constant on average with increasing *NPC* while recovering the fast simulated period. The Enright method returns on average, better results than the Autocorrelation or DFT methods. The Autocorrelation method is the least accurate, producing average differences starting around 60% and decreasing to 35% with 20 *NPC*. (B) The DFT method produces the most accurate results, but overall, average differences are approximately 30%. The red diamond, the green square and the blue circle represent the Autocorrelation, Enright and DFT methods, respectively.

We investigated the ability of the Autocorrelation, Enright and DFT methods to recover periods in more complex data sets, namely bi-oscillatory data (see Supplementary Fig. 1B). When comparing both simulated periods with the periods returned by methods using the minimum significant p-value, we found very different outcomes when examining our ability to recover fast versus slow simulated periods in the data (see Fig. 6 and Fig. 7).

Both the Enright and DFT method's *RD* slightly decreased with increasing *NC* in the estimation of the fast simulated period. Nevertheless, the Enright method returned, on average, better results than the DFT method (Fig. 6A). With 3 *NC*, the DFT and Enright methods recovered periods having relative differences of 20% and 5%, respectively and both methods decayed at a similar rate with increasing *NC*. With 10 *NC*, the Enright method recovered the fast simulated periods with a precision close to optimal. The Autocorrelation method performed far worse, as we observed relative differences close to 40%; this value remained approximately constant with increasing *NC* (Fig. 6A). While the Autocorrelation method performed similarly in recovering the slow period of bi-oscillatory data, the Enright method relative differences jumped to close to 50% and were relatively constant across *NC* (see Fig. 6B). The slow period was best recovered by the DFT method, although the average *RD* is close to 30%.

While recovering the fast simulated period, the Enright and DFT method's performance remained on average relatively unchanged as the number of points per cycle (*NPC*) was increased (Fig. 7A). The Enright method was the most accurate, never surpassing 2.5%, followed by the DFT method that had accuracies values of 15% The Autocorrelation method provided low accuracy, greater than 30% *RD*, although this decreased as *NPC* was increased (Fig. 7A). A different picture emerged from the average relative differences obtained as a function of *NPC* while recovering the slow simulated period. As with the number of cycles, the slow period is best recovered by the DFT method, but overall, the simulated period was poorly estimated (the *RD* were approximately 30%) (Fig. 7B).

## Discussion

The Autocorrelation, Enright and DFT methods are widely used in the life sciences and other scientific fields. To investigate how well these methods recovered oscillatory periods, we applied all three methods to mono and bi-oscillatory simulated course data having different number of cycles (*NC*) as well as different number of points per cycle (*NPC*). On the periodograms and frequency spectra obtained for each data set, we only considered extrema values higher than 10% of the maximum value. This eliminated candidates that had very low peaks, which were unlikely to be significant. Both Autocorrelation and Enright methods cannot distinguish the fundamental frequency from its associated harmonics. For this reason, we also removed all multiples of a candidate period in their respective periodograms. There was no need to remove multiples of a period using the DFT method. For example, a peak frequency of 0.1667 (period 6) would not show up if a real period of 3 was present in the data. To evaluate the significance of a computationally feasible number of candidate periods, we evaluated the significance of less than 5 candidate periods. While this was an arbitrary threshold, it guaranteed high accuracy when analyzing mono-oscillatory data or the fast period of bi-oscillatory data. We assumed that the noise had a Gaussian distribution and followed Eq. (3). However, this noise distribution only accounts for amplitude fluctuations and does not describe period fluctuations. The results of our analysis could vary under different noise functions. This is an issue that requires further investigation, but it is outside of the scope of the current study.

Besides simply identifying a period, one would like to know if a period is significant or was obtained by chance. This is especially important for complex data sets, containing high levels of noise - or more than one oscillatory period. The significance of oscillatory periods is often associated with theoretical false alarm probability functions that have been the subject of debate [14], [15]. This is the case because these functions are only applicable under limited conditions and often fail to provide an adequate quantitative measure of the probability of obtaining a particular period [16]. In this paper, instead of relying in false alarm probability functions, we computed the significance of the oscillatory periods by computing random permutations of the time course data. Since the period is obtained by identifying local maxima in the periodogram and frequency spectra, we ascertained the significance of a period measurement by calculating the probability of obtaining a local maximum after several random data permutations. Since our goal was to determine the minimum number of cycles and number of points required to accurately estimate significant periods, it is important that our method can determine the significance of an extracted period without being limited by the size of the data set being sampled.

In the present study, simulations of oscillatory sine waves provided useful quantitative information as to how to best design an experiment to estimate the periods of oscillations using the Autocorrelation, Enright, and DFT methods. Our analysis suggests that the optimal candidate period of an oscillation is the one associated with a minimum p-value, rather than the highest peak of a periodogram or frequency spectrum. This is especially true for more complex data when more than one period may be present in the data. We also found that the Enright method was the most accurate in estimating the period of mono-oscillatory data or the fast period of bi-oscillatory data. The method was effective when experimental noise ranged up to 60% (Fig. 3 and Fig. 4). Importantly, the accurate estimation of these periods required the collection of time course data having at least five oscillatory cycles (*NC≥5*) and four data points per cycle (*NPC≥4*) (Fig. 5, Fig. 6 and Fig. 7). These optimal characteristics are all present in the recordings of pancreatic islet free calcium oscillations presented in Fig. 2. This explains why we were able to identify these calcium oscillations periods successfully.

## Supporting Information

Figure S1 **Representation of mono and bi-oscillatory data**. (A) Oscillatory data containing three cycles of one single period. The first cycle is represented as ‘Period - Cycle 1’ and the second cycle is represented as ‘Period - Cycle 2’. Each cycle is made of 15 points. (B) Oscillatory data containing five cycles of a fast period and three cycles of a slow period to form a bi-oscillatory data. The first two cycles of the fast period are indicated as ‘Fast Period Cycle 1’ and ‘Fast Period Cycle 2’. The first two cycles of the slow period are indicated as ‘Slow Period Cycle 1’ and ‘Slow Period Cycle 2’.
(TIF)

File S1 **MATLAB codes for Autocorrelation, Enright and DFT methods implemented in this paper.**
(PDF)

File S2 **Sample data of islet Ca2+ oscillations.** Source of data: *PLoS ONE* 7: e34036.
(CSV)
