# Supplementary figures and images for: Optimal Experimental Design to Estimate Statistically Significant Periods of Oscillations in Time Course Data
Source: PLoS One. 2014 Apr 3;9(4):e93826. doi: 10.1371/journal.pone.0093826 (PMC3974819; doi:10.1371/journal.pone.0093826)

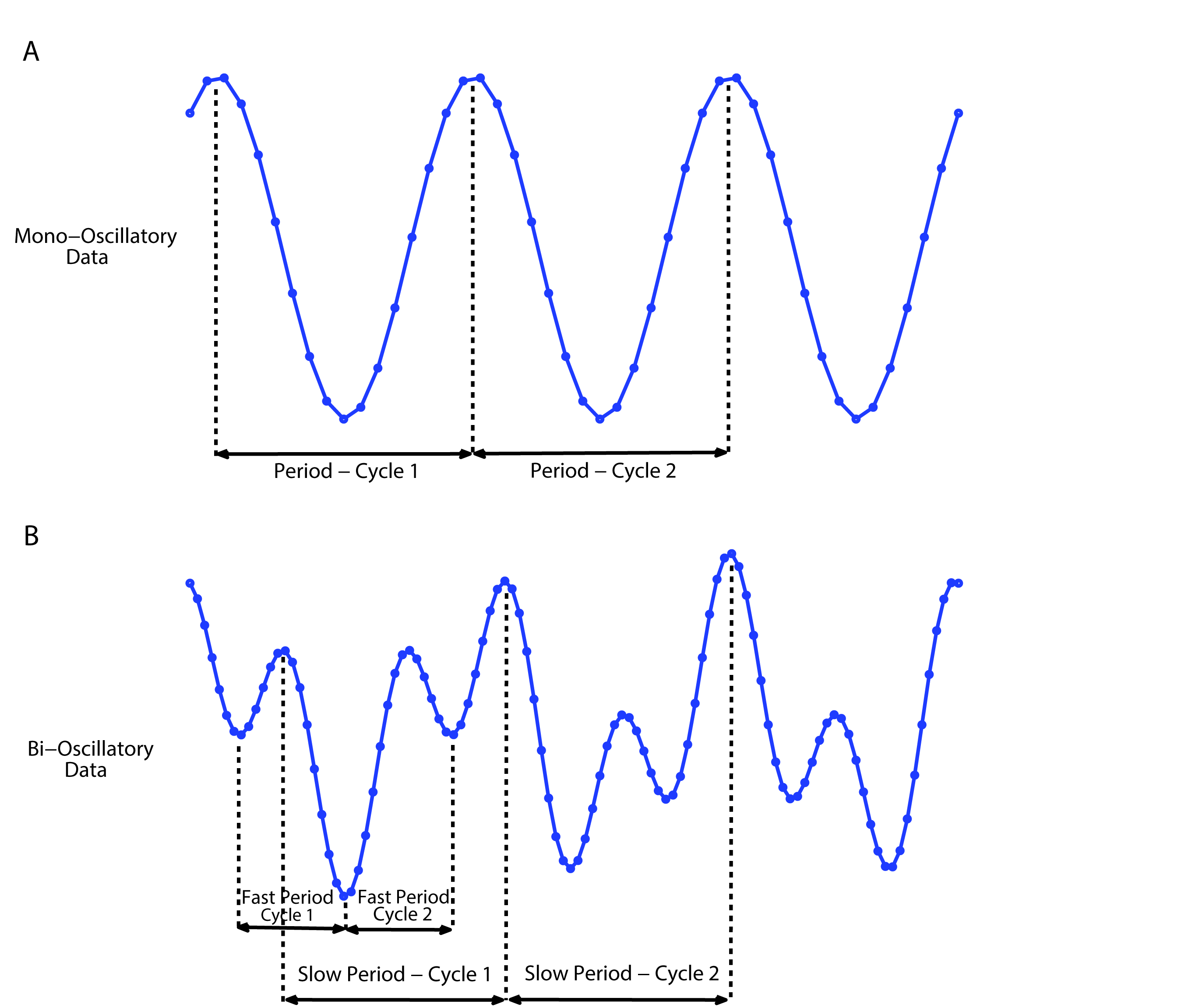

Supplement: Figure S1 — Representation of mono and bi-oscillatory data. (A) Oscillatory data containing three cycles of one single period. The first cycle is represented as ‘Period - Cycle 1’ and the second cycle is represented as ‘Period - Cycle 2’. Each cycle is made of 15 points. (B) Oscillatory data containing five cycles of a fast period and three cycles of a slow period to form a bi-oscillatory data. The first two cycles of the fast period are indicated as ‘Fast Period Cycle 1’ and ‘Fast Period Cycle 2’. The first two cycles of the slow period are indicated as ‘Slow Period Cycle 1’ and ‘Slow Period Cycle 2’. (TIF) [file pone.0093826.s001.tif]
