# Supplementary material for: Optimal Experimental Design to Estimate Statistically Significant Periods of Oscillations in Time Course Data
Source: PLoS One. 2014 Apr 3;9(4):e93826. doi: 10.1371/journal.pone.0093826 (PMC3974819; doi:10.1371/journal.pone.0093826)
Supplement: File S1 — MATLAB codes for Autocorrelation, Enright and DFT methods implemented in this paper. (PDF) [file pone.0093826.s002.pdf]

-------------------------   Test.m FILE   -------------------------

```
function []=test(ntest)
% This function applies the Autocorrelation, Enright and DFT methods to
% test data. It comprises two different tests:
% Test 1 - Tests in silico data
% Test 2 - Tests experimental calcium oscillations (provided by the file
% data1.csv)
% --------------------------------------------------
% For each method applied to a time course, the test produces 15 different
% values, 3 for each of up to 5 candidate periods requested. The first
% value correspond to the identified period and will be negative if not
% significant (p-value < 0.05). The second and third value correspond to
% the power and p-value obtained for that period, respectively.
% --------------------------------------------------
% To run, just type 'test(1)' or 'test(2)' or 'test([1 2])' in the path
% where all files reside

close all;
clc;
n=0;

% ------------------------------ Test 1 ------------------------------
% Generates in silico periodic data

n=n+1;
if ~isempty(find(ntest==1,1))
    npperiod=15; % Number of points per period
    per=[10]; % Period time
    nperiods=10; % Number of repetitions
    noise=0; % Noise level

[xdata,ydata]=gen_input(ones(1,length(per)),per,nperiods,npperiod,noise);
    [res1 res2 res3]=apply(xdata,ydata,5,[1 2 3],1) % This asks for up to 5
candidate periods
end

% ------------------------------ Test 2 ------------------------------
% Identifies significant periods of Calcium oscilations (as mentioned in
% the paper)

n=n+1;
if ~isempty(find(ntest==2,1))
    display('Test 2');
    data=load('data1.csv');
    [x y]=size(data);
    pos_min=1;
    pos_max=x;
    xdata=data(:,1); % Time data
    for i=2:16 % Each column correspond to a different time course of
calcium concentrations
        str=strcat('----- Analyzing column: ',num2str(i),' in file:
data1.csv', ' -----');
        display(str);
        ydata=data(pos_min:pos_max,i)-
(ones(length(pos_min:pos_max),1)*mean(data(pos_min:pos_max,i)));
        grid on;
        [res1 res2 res3]=apply(xdata,ydata,5,[1 2 3],i-1) % This asks for
```

```
up to 5 candidate periods
    end
end

% --------------------------- Main Function ---------------------------
%


    function [res1 res2 res3]=apply(xdata,ydata,nop,methods,fign)
        for i=1:length(methods)
            method=methods(i);
            if method==1
                display('Applying the Autocorrelation method...');
                res1=autocorrelation(xdata,ydata,nop,10000,'perms',[1
2],fign);
            else
                if method==2
                    display('Applying the Enright Periodogram method...');
                    per_range=max(xdata)-min(xdata);

res2=chi2(xdata,ydata,min(xdata)+floor(per_range/2),nop,10000,'perms',[3],f
ign);
                else
                    display('Applying the FFT method...');
                    per_range=max(xdata)-min(xdata);
                    Fs_aux=length(xdata)/per_range;

res3=dft(xdata,ydata,Fs_aux,floor(per_range/2),nop,10000,'perms',[4],fign);
                end
            end
        end
    end

end
```

----------------------- Autocorrelation.m FILE --------------------------

```
function [res]=autocorrelation(xdata,ydata,noscip,maxperm,func,plts,fign)
% ----------
% func is 'perms' or 'normal'
% maxperm is the number of permutations for the calculation of the null
distribution

n=0; % Plot counter
res=zeros(1,noscip*3); % Final Results

% ----------

n=n+1;
if ~isempty(find(plts==n,1))
    figure(fign);
    subplot(2,2,1);
    plot(xdata,ydata,'k');
    set(gca,'fontweight','b','fontsize',16);
    xlabel('Time (min)','fontweight','b','fontsize',16);
    ylabel('Ca^{2+} ','fontweight','b','fontsize',16);
    grid on;
end

nLags=floor(length(ydata)/2);
[ACF, ~, ~]=autocorr(ydata,nLags);
ACF=ACF(2:end);
xaxis=(1:nLags)*(xdata(2)-xdata(1));

n=n+1;
if ~isempty(find(plts==n,1))
    figure(fign);
    subplot(2,2,2);
    plot(xaxis,ACF,'k');
    set(gca,'fontweight','b','fontsize',16);
    xlabel('Lag (min)','fontweight','b','fontsize',16);
    ylabel('ACF','fontweight','b','fontsize',16);
    grid on;
end

% Find the extrema of the ACF function
[ymax,imax,xxx,yyy]=extrema(ACF);

if ~isempty(ymax)
    % Only consider ACF extrema values higher than 0.1 the maximum ACF
value
    cand=find(ymax>(max(ACF)*0.1));
    ymax=ymax(cand);
    imax=imax(cand);

    % Remove the first point of ACF (always unity)
    cand=find(imax==1);
    imax(cand)=[];
    ymax(cand)=[];
```

```
    % Sort by ascending period
    [imax order]=sort(imax);
    ymax=ymax(order);

    % Remove multiples of periods
    l=1;
    while l<length(imax)
        posits=~mod(imax,imax(l))&imax~=imax(l);
        imax(posits)=[];
        ymax(posits)=[];
        l=l+1;
    end

    % Filter results by the number of oscillation ACF values we are
interested in
    if (length(ymax)>noscip)
        ymax=ymax(1:noscip);
        imax=imax(1:noscip);
    end
end

if ~isempty(ymax)

    if ~isempty(find(plts==2,1))
        figure(fign);
        subplot(2,2,2);
        hold on;
        plot(xaxis(imax),ymax,'ko');
    end

    % Obtain the random permutations
    ydata_aux=zeros(maxperm,length(xdata));
    for m=1:maxperm
        if strcmp(func,'perms')
            perm=randperm(length(xdata));
            ydata_aux(m,:)=ydata(perm);
        elseif strcmp(func,'normal')
            ydata_aux(m,:)=mean(ydata)+std(ydata).*randn(1,length(xdata));
        end
    end

    % Obtain the ACF from random permutations
    ACF_tt=zeros(length(imax),maxperm);
    for m=1:maxperm

[ACF_aux,~,~]=autocorr(ydata_aux(m,:),floor(length(ydata_aux(m,:))/2));
        ACF_aux=ACF_aux(2:end);
        for l=1:length(imax)
            ACF_tt(l,m)=ACF_aux(imax(l));
        end
    end

    % Calculate the significance
    sigpos=[];
    for l=1:length(imax)
        aux=length(find(ACF_tt(l,:)>ymax(l)));
        pvalue=aux/maxperm;
```

```
        index=3*(l-1)+1;
        if (pvalue < 0.05)   % 5% Significance level
            sigpos=[sigpos l];
            res(index)=xdata(imax(l)+1)-xdata(1); % stores the period
        else
            res(index)=(xdata(imax(l)+1)-xdata(1))*-1;
        end
        res(index+1)=ymax(l); % stores the power
        res(index+2)=pvalue; % stores the pvalue
    end

    if ~isempty(find(plts==2,1))
        sprintf('Significant Periods according to random data: ');
        for m=1:length(sigpos)
            figure(fign);
            subplot(2,2,2);
            hold on;
            plot(xaxis(imax(sigpos(m))),ymax(sigpos(m)),'k*');
        end
    end

end

end
```

--------------------------- Chi2.m FILE --------------------------

```
function [res]=chi2(xdata,ydata,max_per,noscip,maxperm,func,plts,fign)
% ----------
% func is 'perms' or 'normal'
% maxperm is the number of permutations for the calculation of the null
distribution

n=0; % Plot counter
chi=0; % Change this variable to 1 if you would want to make chi2
calculations
min_per=min(xdata); % Minimum period to evaluate
res=zeros(1,noscip*3); % Final Results

% ----------

n=n+1;
if ~isempty(find(plts==n,1))
    figure(fign);
    plot(xdata,ydata,'k');
    xlabel('Time');
    ylabel('Value');
end

minpoints=length(find(xdata<=min_per));
maxpoints=length(find(xdata<=max_per));
Qp=calc_Qp(xdata,ydata,minpoints,maxpoints,var(ydata));
xaxis=(minpoints:maxpoints)*(xdata(2)-xdata(1));

n=n+2;
if ~isempty(find(plts==n,3))
    figure(fign);
    subplot(2,2,3);
    plot(xaxis,Qp,'k');
    set(gca,'fontweight','b','fontsize',16);
    xlabel('Period (min)','fontweight','b','fontsize',16);
    ylabel('Qp','fontweight','b','fontsize',16);
    grid on;
end

% n=n+1;
% if ~isempty(find(plts==n,1))
%     figure(n);
%     plot(xdata(minpoints:maxpoints),Qp/maxpoints);
%     xlabel('Period');
%     ylabel('Robustness Qp (Fraction of the maximum possible Qp)');
% end

% ----------

% Plots the maximum Qp with increasing number of repetitions
% n=n+1;
% if ~isempty(find(plts==n,1))
%     figure(n);
%     plot(maxim);
%     xlabel('Number of repetitions');
```

```
%     ylabel('Maximum Qp');
% end

% ----------

% Find the extrema of the Qp function
[ymax,imax,xxx,yyy]=extrema(Qp);

if ~isempty(ymax)
    % Only consider Qp extrema values higher than 0.1 the maximum Qp value
    cand=find(ymax>(max(Qp)*0.1));
    ymax=ymax(cand);
    imax=imax(cand);

    % Remove the last point of xdata if it shows up
    cand=find(imax==length(xdata));
    imax(cand)=[];
    ymax(cand)=[];

    % Sort by ascending period
    [imax order]=sort(imax);
    ymax=ymax(order);

    % Remove multiples of periods
    l=1;
    while l<length(imax)
        posits=~mod(imax,imax(l))&imax~=imax(l);
        imax(posits)=[];
        ymax(posits)=[];
        l=l+1;
    end

    % Filter results by the number of oscillation Qp values we are
interested in
    if (length(ymax)>noscip)
        ymax=ymax(1:noscip);
        imax=imax(1:noscip);
    end
end

if ~isempty(ymax)
    if ~isempty(find(plts==3,1))
        figure(fign);
        subplot(2,2,3);
        hold on;
        plot(xaxis(imax),ymax,'ko');
    end

    % Obtain the random permutations
    ydata_aux=zeros(maxperm,length(xdata));
    for m=1:maxperm
        if strcmp(func,'perms')
            perm=randperm(length(xdata));
            ydata_aux(m,:)=ydata(perm);
        elseif strcmp(func,'normal')
            ydata_aux(m,:)=mean(ydata)+std(ydata).*randn(1,length(xdata));
```

```
        end
    end

    % Calculate the significance
    sigpos=[];
    variance=var(ydata);
    for l=1:length(imax)
        minpoints=imax(l);
        maxpoints=minpoints;
        Qp_tt=zeros(1,maxperm);
        for m=1:maxperm

Qp_tt(m)=calc_Qp(xdata,ydata_aux(m,:),minpoints,maxpoints,variance);
        end
        aux=length(find(Qp_tt>ymax(l)));
        pvalue=aux/maxperm;
        index=3*(l-1)+1;
        if (pvalue < 0.05)   % 5% Significance level
            sigpos=[sigpos l];
            res(index)=xdata(imax(l)+1)-xdata(1);
        else
            res(index)=(xdata(imax(l)+1)-xdata(1))*-1;
        end
        res(index+1)=ymax(l); % stores the power
        res(index+2)=pvalue; % stores the pvalue
    end

    if ~isempty(find(plts==3,1))
        sprintf('Significant Periods according to random data: ');
        for m=1:length(sigpos)
            figure(fign);
            subplot(2,2,3);
            hold on;
            plot(xaxis(imax(sigpos(m))),ymax(sigpos(m)),'k*');
        end
    end

end

% ----------

% This function calculates the Qp statistic value using the chi-square
% periodogram for time series data
    function Qp = calc_Qp(x,y,minpoints,maxpoints,variance)
        len_Qp=maxpoints-minpoints+1;
        Qp=zeros(1,len_Qp);

        npoints=minpoints:maxpoints;
        for k=1:length(npoints)
            nblocks=floor(length(x)/npoints(k));
            table=zeros(nblocks,npoints(k));
            for i=1:nblocks
                for j=1:npoints(k)
                    pos=j+npoints(k)*(i-1);
                    table(i,j)=y(pos);
                end
            end
```

```
            if (len_Qp==1)
                Qp=npoints(k)*nblocks*var(mean(table,1))/variance;
            else
                Qp(k)=npoints(k)*nblocks*var(mean(table,1))/variance;
            end
        end
    end

% ----------

end
```

--------------------------   DFT.m FILE   -------------------------

```
function res=dft(xdata,ydata,fs,max_per,noscip,maxperm,func,plts,fign)
% ----------
% func is 'perms' or 'normal'
% maxperm is the number of permutations for the calculation of the null
distribution

n=0;
res=zeros(1,noscip*3); % Final Results

% ----------

n=n+1;
if ~isempty(find(plts==n,1))
    figure(fign);
    plot(xdata,ydata,'k');
    xlabel('Time');
    ylabel('Value');
end

[f mx]=calc_fft(ydata,fs,max_per);

n=n+3;
if ~isempty(find(plts==n,1))
    figure(fign);
    subplot(2,2,4);
    plot(f,mx,'k');
    set(gca,'fontweight','b','fontsize',16);
    xlabel('Frequency (cycles per min)','fontweight','b','fontsize',16);
    ylabel('Power','fontweight','b','fontsize',16);
    grid on;
end

% Find the extrema and sort by the highest power (descencent)
[ymax,imax,xxx,yyy]=extrema(mx);

if ~isempty(ymax)
    % Only consider extrema Power values higher than 0.1 the maximum Qp
value
    cand=find(ymax>(max(mx)*0.1));
    ymax=ymax(cand);
    imax=imax(cand);

    % Filter results by the number of oscillation powers we are interested
in
    if (length(ymax)>noscip)
        ymax=ymax(1:noscip);
        imax=imax(1:noscip);
    end

    % Remove zero frequencies if they exist
    freqs=find(f(imax)==0);
    imax(freqs)=[];
    ymax(freqs)=[];
```

```
%     % Sort results by the highest frequencies (lower periods)
%     [sr1 sr2]=sort(f(imax),'descend');
%     ymax=ymax(sr2);
%     imax=imax(sr2);
end

if ~isempty(ymax)

    if ~isempty(find(plts==4,1))
        figure(fign);
        subplot(2,2,4);
        hold on;
        plot(f(imax),ymax,'ko');
    end

    % ---------------

    count=0;
    for m=1:maxperm
        if strcmp(func,'perms')
            perm=randperm(length(xdata));
            ydata_aux=ydata(perm);
        elseif strcmp(func,'normal')
            ydata_aux=mean(ydata)+std(ydata).*randn(1,length(xdata));
        end
        [f_perm mx_perm]=calc_fft(ydata_aux,fs,max_per);
        [ymax_perm,imax_perm,xxx,yyy]=extrema(mx_perm);
        if ~isempty(ymax_perm)
            if ~(f_perm(imax_perm(1))==0)
                count=count+1;
                pow(count)=ymax_perm(1); % I am using the biggest power in
the spectra
            end
        end
    end

    % ---------------

    sigpos=[];
    for n=1:length(ymax)
        aux=length(find(pow>ymax(n)));
        pvalue=aux/count;
        index=3*(n-1)+1;
        if (pvalue < 0.05)   % 5% Significance level
            sigpos=[sigpos n];
            res(index)=1/f(imax(n));
        else
            res(index)=(1/f(imax(n)))*-1;
        end
        res(index+1)=ymax(n); % stores the power
        res(index+2)=pvalue; % stores the pvalue
    end

    if ~isempty(find(plts==4,1))
        sprintf('Significant Periods according to random data: ');
        if ~isempty(sigpos)
```

```
            figure(fign);
            subplot(2,2,4);
            hold on;
            plot(f(imax(sigpos)),ymax(sigpos),'k*');
        end
    end
end

% ----------

% This function calculates the frequency and the period of potential
% oscillations using the discrete fourier transform
    function [f,MX] = calc_fft(x,Fs,max_per)
        Fn=Fs/2;                                % Nyquist frequency
        NFFT=2.^(ceil(log(length(x))/log(2)));  % Next highest power of 2
                                                % greater than length(x).
        [MX,f]=periodogram(x,[],NFFT,Fs);       % The default window is
used
        ind_lim=find(f>1/max_per);              % This imposes a lower
limit
        f=f(ind_lim);                           % limit on the frequency
        MX=MX(ind_lim);
    end
end
```

```
---------------------------   gen_input.m FILE   --------------------------

function [xdata,ydata]=gen_input(amps,pers,nperiods,npperiod,noise)
% This function generates the input data
ydata=0;
for i=1:length(pers)
    freq=1/pers(i); % Frequency
    xdata=1:min(pers)/npperiod:(max(pers)*nperiods)+1;
    ydata=ydata+amps(i)*sin(2*pi*xdata*freq); % Create a sine wave...
end
ydata=ydata+noise.*randn(1,length(xdata));
end
% ----------
```

--------------------------- extrema.m FILE --------------------------

```
function [xmax,imax,xmin,imin] = extrema(x)
%EXTREMA   Gets the global extrema points from a time series.
%   [XMAX,IMAX,XMIN,IMIN] = EXTREMA(X) returns the global minima and maxima
%   points of the vector X ignoring NaN's, where
%    XMAX - maxima points in descending order
%    IMAX - indexes of the XMAX
%    XMIN - minima points in descending order
%    IMIN - indexes of the XMIN
%
%   DEFINITION (from http://en.wikipedia.org/wiki/Maxima_and_minima):
%   In mathematics, maxima and minima, also known as extrema, are points in
%   the domain of a function at which the function takes a largest value
%   (maximum) or smallest value (minimum), either within a given
%   neighbourhood (local extrema) or on the function domain in its entirety
%   (global extrema).
%
%   Example:
%      x = 2*pi*linspace(-1,1);
%      y = cos(x) - 0.5 + 0.5*rand(size(x)); y(40:45) = 1.85; y(50:53)=NaN;
%      [ymax,imax,ymin,imin] = extrema(y);
%      plot(x,y,x(imax),ymax,'g.',x(imin),ymin,'r.')
%
%   See also EXTREMA2, MAX, MIN

%   Written by
%   Lic. on Physics Carlos Adri·n Vargas Aguilera
%   Physical Oceanography MS candidate
%   UNIVERSIDAD DE GUADALAJARA
%   Mexico, 2004
%
%   nubeobscura@hotmail.com

% From       : http://www.mathworks.com/matlabcentral/fileexchange
% File ID    : 12275
% Submited at: 2006-09-14
% 2006-11-11 : English translation from spanish.
% 2006-11-17 : Accept NaN's.
% 2007-04-09 : Change name to MAXIMA, and definition added.


xmax = [];
imax = [];
xmin = [];
imin = [];

% Vector input?
Nt = numel(x);
if Nt ~= length(x)
 error('Entry must be a vector.')
end

% NaN's:
inan = find(isnan(x));
```

```
indx = 1:Nt;
if ~isempty(inan)
 indx(inan) = [];
 x(inan) = [];
 Nt = length(x);
end

% Difference between subsequent elements:
dx = diff(x);

% Is an horizontal line?
if ~any(dx)
 return
end

% Flat peaks? Put the middle element:
a = find(dx~=0);              % Indexes where x changes
lm = find(diff(a)~=1) + 1;    % Indexes where a do not changes
d = a(lm) - a(lm-1);          % Number of elements in the flat peak
a(lm) = a(lm) - floor(d/2);   % Save middle elements
a(end+1) = Nt;

% Peaks?
xa  = x(a);             % Serie without flat peaks
b = (diff(xa) > 0);     % 1  =>  positive slopes (minima begin)
                        % 0  =>  negative slopes (maxima begin)
xb  = diff(b);          % -1 =>  maxima indexes (but one)
                        % +1 =>  minima indexes (but one)
imax = find(xb == -1) + 1; % maxima indexes
imin = find(xb == +1) + 1; % minima indexes
imax = a(imax);
imin = a(imin);

nmaxi = length(imax);
nmini = length(imin);

% Maximum or minumim on a flat peak at the ends?
if (nmaxi==0) && (nmini==0)
 if x(1) > x(Nt)
  xmax = x(1);
  imax = indx(1);
  xmin = x(Nt);
  imin = indx(Nt);
 elseif x(1) < x(Nt)
  xmax = x(Nt);
  imax = indx(Nt);
  xmin = x(1);
  imin = indx(1);
 end
 return
end

% Maximum or minumim at the ends?
if (nmaxi==0)
 imax(1:2) = [1 Nt];
elseif (nmini==0)
 imin(1:2) = [1 Nt];
```

```
else
 if imax(1) < imin(1)
  imin(2:nmini+1) = imin;
  imin(1) = 1;
 else
  imax(2:nmaxi+1) = imax;
  imax(1) = 1;
 end
 if imax(end) > imin(end)
  imin(end+1) = Nt;
 else
  imax(end+1) = Nt;
 end
end
xmax = x(imax);
xmin = x(imin);

% NaN's:
if ~isempty(inan)
 imax = indx(imax);
 imin = indx(imin);
end

% Same size as x:
imax = reshape(imax,size(xmax));
imin = reshape(imin,size(xmin));

% Descending order:
[temp,inmax] = sort(-xmax); clear temp
xmax = xmax(inmax);
imax = imax(inmax);
[xmin,inmin] = sort(xmin);
imin = imin(inmin);


% Carlos Adri·n Vargas Aguilera. nubeobscura@hotmail.com
```

-------------------------- autocorr.m FILE -------------------------

```
function varargout = autocorr(Series , nLags , Q , nSTDs)
%AUTOCORR Compute or plot sample auto-correlation function.
%   Compute or plot the sample auto-correlation function (ACF) of a
univariate,
%   stochastic time series. When called with no output arguments, AUTOCORR
%   displays the ACF sequence with confidence bounds.
%
%   [ACF, Lags, Bounds] = autocorr(Series)
%   [ACF, Lags, Bounds] = autocorr(Series , nLags , M , nSTDs)
%
%   Optional Inputs: nLags , M , nSTDs
%
% Inputs:
%   Series - Vector of observations of a univariate time series for which
the
%     sample ACF is computed or plotted. The last row of Series contains
the
%     most recent observation of the stochastic sequence.
%
% Optional Inputs:
%   nLags - Positive, scalar integer indicating the number of lags of the
ACF
%     to compute. If empty or missing, the default is to compute the ACF at
%     lags 0,1,2, ... T = minimum[20 , length(Series)-1]. Since an ACF is
%     symmetric about zero lag, negative lags are ignored.
%
%   M - Non-negative integer scalar indicating the number of lags beyond
which
%     the theoretical ACF is deemed to have died out. Under the hypothesis
that
%     the underlying Series is really an MA(M) process, the large-lag
standard
%     error is computed (via Bartlett's approximation) for lags > M as an
%     indication of whether the ACF is effectively zero beyond lag M. On
the
%     assumption that the ACF is zero beyond lag M, Bartlett's
approximation
%     is used to compute the standard deviation of the ACF for lags > M. If
M
%     is empty or missing, the default is M = 0, in which case Series is
%     assumed to be Gaussian white noise. If Series is a Gaussian white
noise
%     process of length N, the standard error will be approximately
1/sqrt(N).
%     M must be less than nLags.
%
%   nSTDs - Positive scalar indicating the number of standard deviations of
the
%     sample ACF estimation error to compute assuming the theoretical ACF
of
%     Series is zero beyond lag M. When M = 0 and Series is a Gaussian
white
%     noise process of length N, specifying nSTDs will result in confidence
%     bounds at +/-(nSTDs/sqrt(N)). If empty or missing, default is nSTDs =
2
%     (i.e., approximate 95% confidence interval).
```

```
%
% Outputs:
%   ACF - Sample auto-correlation function of Series. ACF is a vector of
%     length nLags + 1 corresponding to lags 0,1,2,...,nLags. The first
%     element of ACF is unity (i.e., ACF(1) = 1 = lag 0 correlation).
%
%   Lags - Vector of lags corresponding to ACF (0,1,2,...,nLags).
%
%   Bounds - Two element vector indicating the approximate upper and lower
%     confidence bounds assuming that Series is an MA(M) process. Note that
%     Bounds is approximate for lags > M only.
%
% Example:
%   Create an MA(2) process from a sequence of 1000 Gaussian deviates, then
%   visually assess whether the ACF is effectively zero for lags > 2:
%
%     randn('state',0)                % Start from a known state.
%     x = randn(1000,1);              % 1000 Gaussian deviates ~ N(0,1).
%     y = filter([1 -1 1] , 1 , x);  % Create an MA(2) process.
%     autocorr(y , [] , 2)            % Inspect the ACF with 95% confidence.
%
% See also CROSSCORR, PARCORR, FILTER.

%   Copyright 1999-2003 The MathWorks, Inc.
%   $Revision: 1.6.2.1 $  $Date: 2003/05/08 21:45:15 $


%
% Reference:
%   Box, G.E.P., Jenkins, G.M., Reinsel, G.C., "Time Series Analysis:
%     Forecasting and Control", 3rd edition, Prentice Hall, 1994.


%
% Ensure the sample data is a VECTOR.
%

[rows , columns]  =  size(Series);

if (rows ~= 1) & (columns ~= 1)
   error('GARCH:autocorr:NonVectorInput' , ' Input ''Series'' must be a
vector.');
end

rowSeries   =  size(Series,1) == 1;

Series      =  Series(:);       % Ensure a column vector
n           =  length(Series);  % Sample size.
defaultLags =  20;              % BJR recommend about 20 lags for ACFs.


%
% Ensure the number of lags, nLags, is a positive
% integer scalar and set default if necessary.
%

if (nargin >= 2) & ~isempty(nLags)
  if prod(size(nLags)) > 1
     error('GARCH:autocorr:NonScalarLags' , ' Number of lags ''nLags'' must
```

```
be a scalar.');
  end
  if (round(nLags) ~= nLags) | (nLags <= 0)
     error('GARCH:autocorr:NonPositiveInteger' , ' Number of lags ''nLags''
must be a positive integer.');
  end
  if nLags > (n - 1)
     error('GARCH:autocorr:LagsTooLarge' , ' Number of lags ''nLags'' must
not exceed ''Series'' length - 1.');
  end
else
  nLags  =  min(defaultLags , n - 1);
end

%
% Ensure the hypothesized number of lags, Q, is a non-negative integer
% scalar, and set default if necessary.
%
if (nargin >= 3) & ~isempty(Q)
  if prod(size(Q)) > 1
     error('GARCH:autocorr:NonScalarQ' , ' Number of lags ''Q'' must be a
scalar.');
  end
  if (round(Q) ~= Q) | (Q < 0)
     error('GARCH:autocorr:NegativeInteger' , ' Number of lags ''Q'' must
be a non-negative integer.');
  end
  if Q >= nLags
     error('GARCH:autocorr:QTooLarge' , ' ''Q'' must be less than
''nLags''.');
  end
else
  Q  =  0;     % Default is 0 (Gaussian white noise hypothisis).
end

%
% Ensure the number of standard deviations, nSTDs, is a positive
% scalar and set default if necessary.
%

if (nargin >= 4) & ~isempty(nSTDs)
  if prod(size(nSTDs)) > 1
     error('GARCH:autocorr:NonScalarSTDs' , ' Number of standard deviations
''nSTDs'' must be a scalar.');
  end
  if nSTDs < 0
     error('GARCH:autocorr:NegativeSTDs' , ' Number of standard deviations
''nSTDs'' must be non-negative.');
  end
else
  nSTDs =  2;     % Default is 2 standard errors (95% condfidence
interval).
end

%
% Convolution, polynomial multiplication, and FIR digital filtering are
% all the same operation. The FILTER command could be used to compute
```

```
% the ACF (by computing the correlation by convolving the de-meaned
% Series with a flipped version of itself), but FFT-based computation
% is significantly faster for large data sets.
%
% The ACF computation is based on Box, Jenkins, Reinsel, pages 30-34, 188.
%

nFFT =  2^(nextpow2(length(Series)) + 1);
F    =  fft(Series-mean(Series) , nFFT);
F    =  F .* conj(F);
ACF  =  ifft(F);
ACF  =  ACF(1:(nLags + 1));         % Retain non-negative lags.
ACF  =  ACF ./ ACF(1);     % Normalize.
ACF  =  real(ACF);

%
% Compute approximate confidence bounds using the Box-Jenkins-Reinsel
% approach, equations 2.1.13 and 6.2.2, on pages 33 and 188, respectively.
%

sigmaQ  =  sqrt((1 + 2*(ACF(2:Q+1)'*ACF(2:Q+1)))/n);
bounds  =  sigmaQ * [nSTDs ; -nSTDs];
Lags    =  [0:nLags]';

if nargout == 0                     % Make plot if requested.

%
%  Plot the sample ACF.
%
  lineHandles  =  stem(Lags , ACF , 'filled' , 'r-o');
  set   (lineHandles(1) , 'MarkerSize' , 4)
  grid  ('on')
  xlabel('Lag')
  ylabel('Sample Autocorrelation')
  title ('Sample Autocorrelation Function (ACF)')
  hold  ('on')
%
%  Plot the confidence bounds under the hypothesis that the underlying
%  Series is really an MA(Q) process. Bartlett's approximation gives
%  an indication of whether the ACF is effectively zero beyond lag Q.
%  For this reason, the confidence bounds (horizontal lines) appear
%  over the ACF ONLY for lags GREATER than Q (i.e., Q+1, Q+2, ... nLags).
%  In other words, the confidence bounds enclose ONLY those lags for
%  which the null hypothesis is assumed to hold.
%

  plot([Q+0.5 Q+0.5 ; nLags nLags] , [bounds([1 1]) bounds([2 2])] , '-b');

  plot([0 nLags] , [0 0] , '-k');
  hold('off')
  a  =  axis;
  axis([a(1:3) 1]);

else

%
```

```
%  Re-format outputs for compatibility with the SERIES input. When SERIES
is
%  input as a row vector, then pass the outputs as a row vectors; when
SERIES
%  is a column vector, then pass the outputs as a column vectors.
%
  if rowSeries
     ACF     =  ACF.';
     Lags    =  Lags.';
     bounds  =  bounds.';
  end

  varargout  =  {ACF , Lags , bounds};

end
```
